# Abrogating PDK4 activates autophagy-dependent ferroptosis in breast cancer via ASK1/JNK pathway

**DOI:** 10.1007/s00432-024-05748-9

**Published:** 2024-04-27

**Authors:** Wenbiao Shi, Jian Wang, Jianbin Chen, Xiaoyan Jin, Yuanfan Wang, Linjun Yang

**Affiliations:** Department of Surgical Oncology, Taizhou Municipal Hospital, No.381 Zhongshan East Road, Jiaojiang District, Taizhou City, 318000 Zhejiang Province China

**Keywords:** ASK1/JNK pathway, Autophagy, Breast cancer, Ferroptosis, PDK4

## Abstract

**Background:**

Targeting ferroptosis mediated by autophagy presents a novel therapeutic approach to breast cancer, a mortal neoplasm on the global scale. Pyruvate dehydrogenase kinase isozyme 4 (PDK4) has been denoted as a determinant of breast cancer metabolism. The target of this study was to untangle the functional mechanism of PDK4 in ferroptosis dependent on autophagy in breast cancer.

**Methods:**

RT-qPCR and western blotting examined PDK4 mRNA and protein levels in breast cancer cells. Immunofluorescence staining appraised light chain 3 (LC3) expression. Fe (2 +) assay estimated total iron level. Relevant assay kits and C11-BODIPY (591/581) staining evaluated lipid peroxidation level. DCFH-DA staining assayed intracellular reactive oxygen species (ROS) content. Western blotting analyzed the protein levels of autophagy, ferroptosis and apoptosis-signal-regulating kinase 1 (ASK1)/c-Jun N-terminal kinase (JNK) pathway-associated proteins.

**Results:**

PDK4 was highly expressed in breast cancer cells. Knockdown of PDK4 induced the autophagy of breast cancer cells and 3-methyladenine (3-MA), an autophagy inhibitor, countervailed the promoting role of PDK4 interference in ferroptosis in breast cancer cells. Furthermore, PDK4 knockdown activated ASK1/JNK pathway and ASK1 inhibitor (GS-4997) partially abrogated the impacts of PDK4 absence on the autophagy and ferroptosis in breast cancer cells.

**Conclusion:**

To sum up, deficiency of PDK4 activated ASK1/JNK pathway to stimulate autophagy-dependent ferroptosis in breast cancer.

**Supplementary Information:**

The online version contains supplementary material available at 10.1007/s00432-024-05748-9.

## Introduction

Breast cancer constitutes one of the most widespread female malignancies accountable for cancer-related deaths (Katsura et al. 2022). According to the global statistics in 2022, breast cancer accounts for almost one-third of newly diagnosed cancer cases among females (Siegel et al. 2022). Breast cancer is widely perceived as a group of heterogeneous diseases that substantially vary at the molecular and clinical level (Aleskandarany et al. 2018; Roulot et al. 2016). Despite the advancements in multimodality therapy combined with surgery, radiotherapy, chemotherapy, endocrine therapy and targeted therapy, alternative treatment approaches are absent to date (Lau et al. 2022). Regulated cell death (RCD), also denoted as programmed cell death (PCD), such as apoptosis, autophagy, and necroptosis, is a natural way in eliminating damaged or abnormal cells implicated in normal cell turnover and tissue homeostasis (Kopeina and Zhivotovsky 2022). Importantly, PCD functions as an essential anticancer defense mechanism (Felici and Piacentini 2015) and autophagic and ferroptotic alternations have been recently recognized as common events during the process of breast cancer (Cocco, et al. 2020; Sui et al. 2022). In this context, in-depth researches should be conducted to study autophagy and ferroptosis underlying the course of breast cancer, thus providing prospective therapeutic targets.

Pyruvate dehydrogenase kinases (PDKs) are critical enzymes located in the mitochondria that phosphorylates the pyruvate dehydrogenase (PDH) to impair the activity of pyruvate dehydrogenase complex (PDC), the key executor in the tricarboxylic acid cycle and glucose metabolism (Golias et al. 2019). Pyruvate dehydrogenase kinase isozyme 4 (PDK4), the predominant isoform of PDK family, has been known to be implicated in plentiful cellular biological behaviors, such as proliferation, invasion, metabolic programming, autophagy, ferroptosis, and so on (Guda et al. 2018; Liu et al. 2021a; Ma et al. 2020; Zhang, et al. 2022). Previous report has mentioned that PDK4 is overexpressed in breast cancer cells and associated with poor patient outcomes (Guda et al. 2018). Further literatures have also supported that PDK4 participates in the aggressive phenotypes and metabolism of breast cancer cells (Dwyer, et al. 2023; Huang et al. 2021). Nonetheless, the impacts of PDK4 on the autophagy and ferroptosis of breast cancer cells are indistinct.

Apoptosis-signal-regulating kinase 1 (ASK1), belonging to the MAPK kinase (MAP3K) family, can serve as an upstream regulatory protein of c-Jun N-terminal kinase (JNK) in response to various stimuli (Flaumenhaft 2017). Emerging studies have evidenced that AKS1/JNK signaling is frequently inactivated in breast cancer to trigger the malignancy (Guo et al. 2016; Zhao et al. 2019; Palit et al. 2015). Moreover, PDK4 may function in human diseases and cancers via regulating ASK1 and JNK pathways (Gao et al. 2022; Lee, et al. 2022).

Accordingly, whether PDK4 functioned in the ferroptosis and autophagy in breast cancer cells via mediating ASK1/JNK pathway was the focus of our present work.

## Materials and methods

### Cultivation and treatment of cells

Non-tumorigenic mammary cell line (MCF-10A) and breast cancer cell lines (MDA-MB-231, SUM190PT) were all supplied by Otwo Biotech (Shenzhen, China). MDA-MB-231 cells were incubated in Leibovitz's L-15 medium (Life Technologies, Karlsruhe, Germany), while other cells were all grown in Roswell Park Memorial Institute (RPMI)-1640 medium (Trace Biosciences, Melbourne, Vic, Australia). In addition, breast cancer MCF-7 cells that were purchased from Typical Culture Preservation Commission Cell Bank, Chinese Academy of Sciences (Shanghai, China) were incubated in minimum essential medium (MEM; Life Technologies, Karlsruhe, Germany). All cells were cultivated in corresponding mediums containing 10% fetal bovine serum (FBS; Trace Biosciences, Melbourne, Vic, Australia) at 37 ℃ with 5% CO_2_.

MCF-7 cells were treated by autophagy inhibitors 3-methyladenine (3-MA; 2.5 mM; Selleck, USA) for 2 h (Cheng et al. 2019) or chloroquine (CQ; 20 μM; Selleck, USA) for 2 h (Shi et al. 2015; Tang et al. 2021) or ASK1 inhibitor (GS-4997; 1 µmol/L; Selleck, USA) for 1 h (Han et al. 2019). Then, small interfering RNA (siRNAs) targeting PDK4 (si-PDK4#1/2) and the scrambled control siRNA (si-NC) that were constructed by Hippo Biotechnology (Huzhou, China) were transfected into cells using XfectTM RNA transfection reagent (Takara, Dalian, China).

### Immunofluorescence (IF) staining

After the immobilization by 4% paraformaldehyde for 30 min and the permeation with 0.5% Triton X-100 for 10 min, MCF-7 cells were blocked with PBS containing 1% BSA. Subsequently, cells were incubated with LC3 antibody (cat. no. #14,600-1-AP; 1/250; Proteintech), LC3B antibody (cat. no. #AF5402; 1/100; Affinity Biosciences), p62 antibody (cat. no. #AF5384; 1/100; Affinity Biosciences) overnight at 4 °C. On the next day, the cells were incubated with secondary antibody conjugated with Alexa Fluor 488 (cat. no. ab150077; 1/200; Abcam) for 1 h at room temperature. The nuclei were stained by 1 mg/ml DAPI. The intensity was recorded under a fluorescence microscope (Leica, Wetzlar, Germany).

### Estimation of total iron level

The total iron content in the cell supernatants was detected using Iron Assay Kit (cat. no. ab83366; Abcam) according to the manufacturer’s instructions. The absorbance of samples was detected at 593 nm under a microplate reader (SLT Lab Instruments GmbH, Salzburg, Austria).

### Measurement of oxidative stress levels

MCF-7 cells were incubated with DCFH-DA probe (10 μmol/l; Elabscience, Shanghai, China) at 37˚C for 30 min in the dark according to the manufacturer’s instructions. Following the wash with PBS, the intensity was detected under a fluorescence microplate reader (BMG Labtech, Offenburg, Germany) with the excitation as 500 nm and the emission as 525 nm.

After the centrifugation at 2000 rpm/min, the activities of malondialdehyde (MDA), 4-hydroxynonenal (4-HNE), superoxide dismutase (SOD) and glutathione peroxidase (GSH-Px) in MCF-7 cells were detected using MDA assay kits (cat. no. J20465; GILED, Wuhan, China), 4-HNE assay kits (cat. no. J21715; GILED, Wuhan, China), SOD assay kits (cat. no. J21118; GILED, Wuhan, China) and GSH-Px assay kits (cat. no. J20841; GILED, Wuhan, China) according to the manufacturer’s instructions. Under a microplate reader, the absorbance was detected at 450 nm.

### C11 BODIPY 581/591 assay

MCF-7 cells were incubated with 5 μmol/L C11-BODIPY^581/591^ probe (Amgicam, Wuhan, China) at 37 ℃ in the dark for 1 h according to the manufacturer’s instructions. Under a fluorescence microscope, the intensity was recorded following PBS washing.

### Reverse transcription-quantitative PCR (RT-qPCR)

Total RNA was prepared from cells using Trizol reagent (Ambion, Austin, TX) according to the manufacturer’s instructions, and then reverse-transcripted into cDNA through ReverTra Ace qPCR RT Kit (TOYOBO Life Science, Shanghai, China). PCR reaction was implemented using THUNDERBIRD^®^ SYBR^®^ qPCR Mix (TOYOBO Life Science, Shanghai, China). PDK4 expression was detected using 2^−ΔΔCq^ approach with GAPDH as a normalizer.

### Western blot

The total proteins were extracted using RIPA buffer (Applygen, Beijing, China) and the protein concentration was detected using BCA method (Applygen, Beijing, China). Following the separation with gel electrophoresis, the proteins were transferred to the PVDF membranes. The membranes were blocked by 5% BSA and then cultivated with primary antibodies targeting PDK4 (CAT. NO. #DF7169; 1/1000), light chain 3B (LC3B; CAT. NO. #AF4650; 1/1000), p62 (CAT. NO. #AF5384; 1/1000), autophagy related 5 (ATG5; CAT. NO. #DF6010; 1/1000), autophagy related 7 (ATG7; CAT. NO. #DF6130; 1/1000), acyl-CoA synthetase long-chain family member 4 (ACSL4; CAT. NO. #DF12141; 1/1000), glutathione peroxidase 4 (GPX4; CAT. NO. #DF6701; 1/1000), ferritin heavy chain 1 (FTH1; CAT. NO. #DF6278; 1/1000), solute carrier family 7a member 11 (SLC7A11; CAT. NO. #DF12509; 1/1000), nuclear receptor coactivator 4 (NCOA4; CAT. NO. #DF4255; 1/1000), apoptosis signal-regulating kinase 1 (ASK1; CAT. NO. #AF6477; 1/1000), phosphorylated apoptosis signal-regulating kinase 1 (p-ASK1; CAT. NO. #AF3477; 1/1000), c-Jun N-terminal kinase (JNK; CAT. NO. #AF6318; 1/1000), phosphorylated c-Jun N-terminal kinase (p-JNK; CAT. NO. #AF3318; 1/1000) and β-actin (CAT. NO. #AF7018; 1/3000) from Affinity Biosciences, followed by the incubation with HRP-linked secondary antibody (CAT. NO. #S0001; 1/3000; Affinity Biosciences). By means of ECL Chemiluminescence solution (Applygen, Beijing, China), the binding signals were scanned.

### Statistics

All data were analyzed using GraphPad Prism 8.01 software (GraphPad Software Inc., CA, USA) and then presented as mean ± standard deviation. Differences among multiple groups were compared using one-way ANOVA followed by Tukey’s post hoc test. P less than 0.05 indicated statistical significance.

## Results

### PDK4 mRNA and protein expression are increased in breast cancer cells

**Fig. 1:**
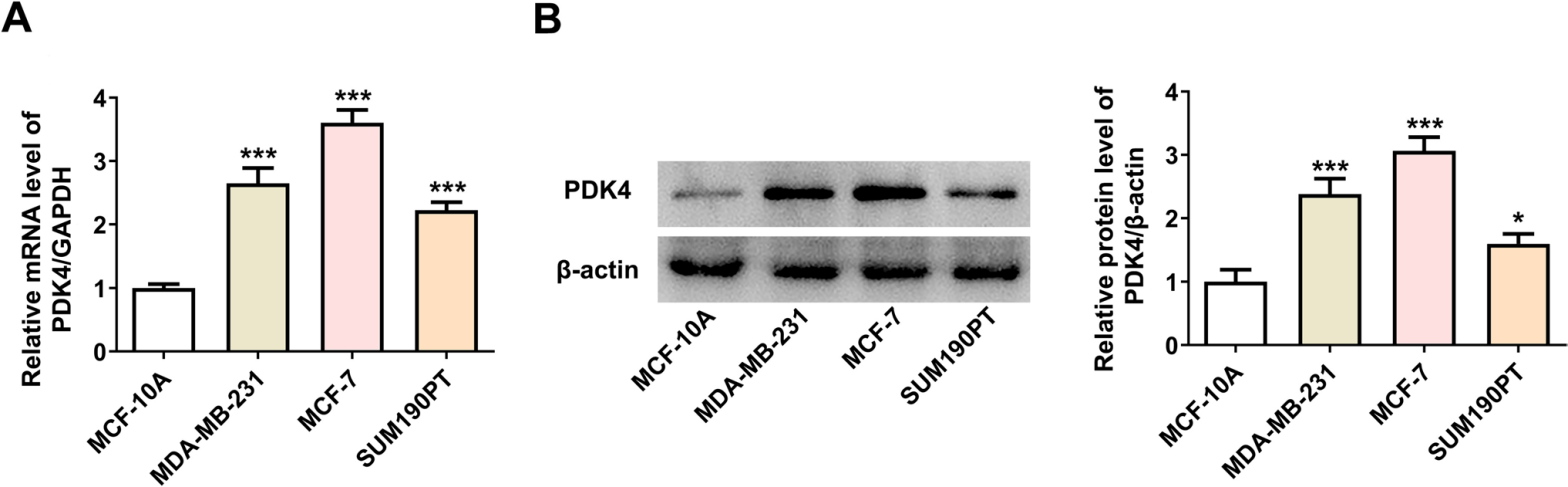
PDK4 mRNA and protein expression are increased in breast cancer cells. **A** RT-qPCR and **B** western blotting analyzed PDK4 mRNA and protein levels in breast cancer cells. Data are presented as mean ± SD. n = 3. ^*^P < 0.05, ^***^P < 0.001 vs. MCF-10A

Before investigating the role of PDK4 in breast cancer, PDK4 expression in breast cancer cells was examined using RT-qPCR and western blotting. It was found that PDK4 expression was noted to be significantly increased at both mRNA and protein level in MDA-MB-231, SUM190PT and MCF-7 cells compared with the non-tumorigenic mammary cell line MCF-10A (Fig. 1A, B). Moreover, the highest PDK4 mRNA and protein levels were observed in MCF-7 cells; therefore, MCF-7 cells were chosen for follow-up experiments.

### Abrogation of PDK4 induces the autophagy of MCF-7 cells

**Fig. 2:**
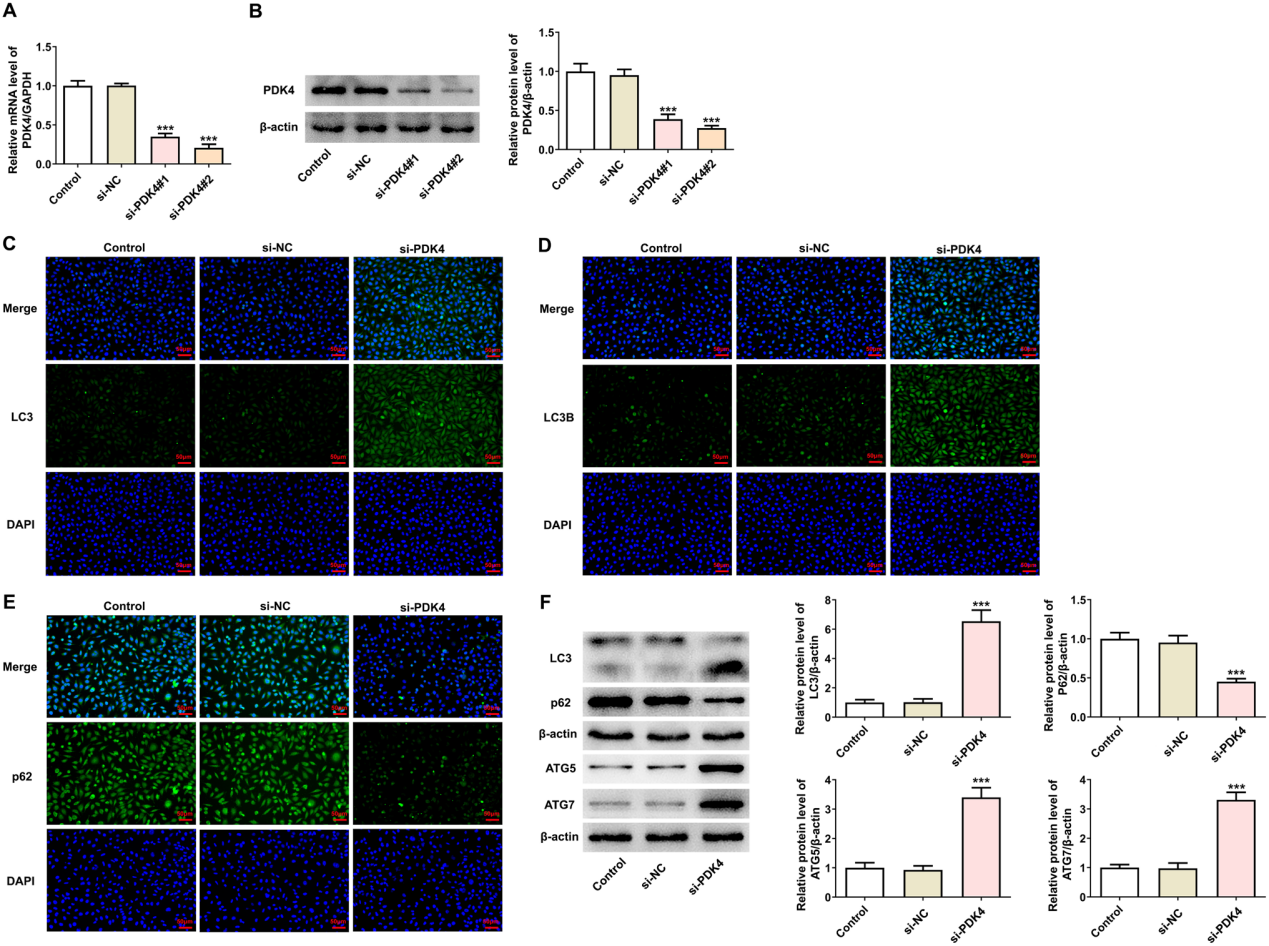
PDK4 silence promotes the autophagy of MCF-7 cells. **A**, **B** Examination of the transfection efficacy of PDK4 interference plasmids in MCF-7 cells by RT-qPCR and western blotting. **C**, **E** Immunofluorescence staining appraised LC3, LC3B and p62 expression. **F** Western blotting tested the protein levels of autophagy-associated proteins. Data are presented as mean ± SD. n = 3. ^***^P < 0.001 vs. si-NC

Considering the overexpression of PDK4 in breast cancer, PDK4 was then knocked down in MCF-7 cells in the following functional experiments. As expected, PDK4 mRNA and protein levels were significantly decreased after the transfection with si-PDK4#1/2 and si-PDK4#2 was chosen for following experiments for its excellent interference efficacy (Fig. 2A, B). As displayed in Fig. 2C, D, immunofluorescence staining presented that when PDK4 was down-regulated, LC3 and LC3B expression was significantly increased whereas p62 expression was decreased. Also, LC3-II/LC3-I, ATG5 and ATG7 protein levels were increased while p62 protein level was decreased in MCF-7 cells transfected with si-PDK4 (Fig. 2F). All these findings hinted the promoting role of PDK4 inhibition in the autophagy in breast cancer cells.

### PDK4 absence activates autophagy-dependent ferroptosis in MCF-7 cells

**Fig. 3:**
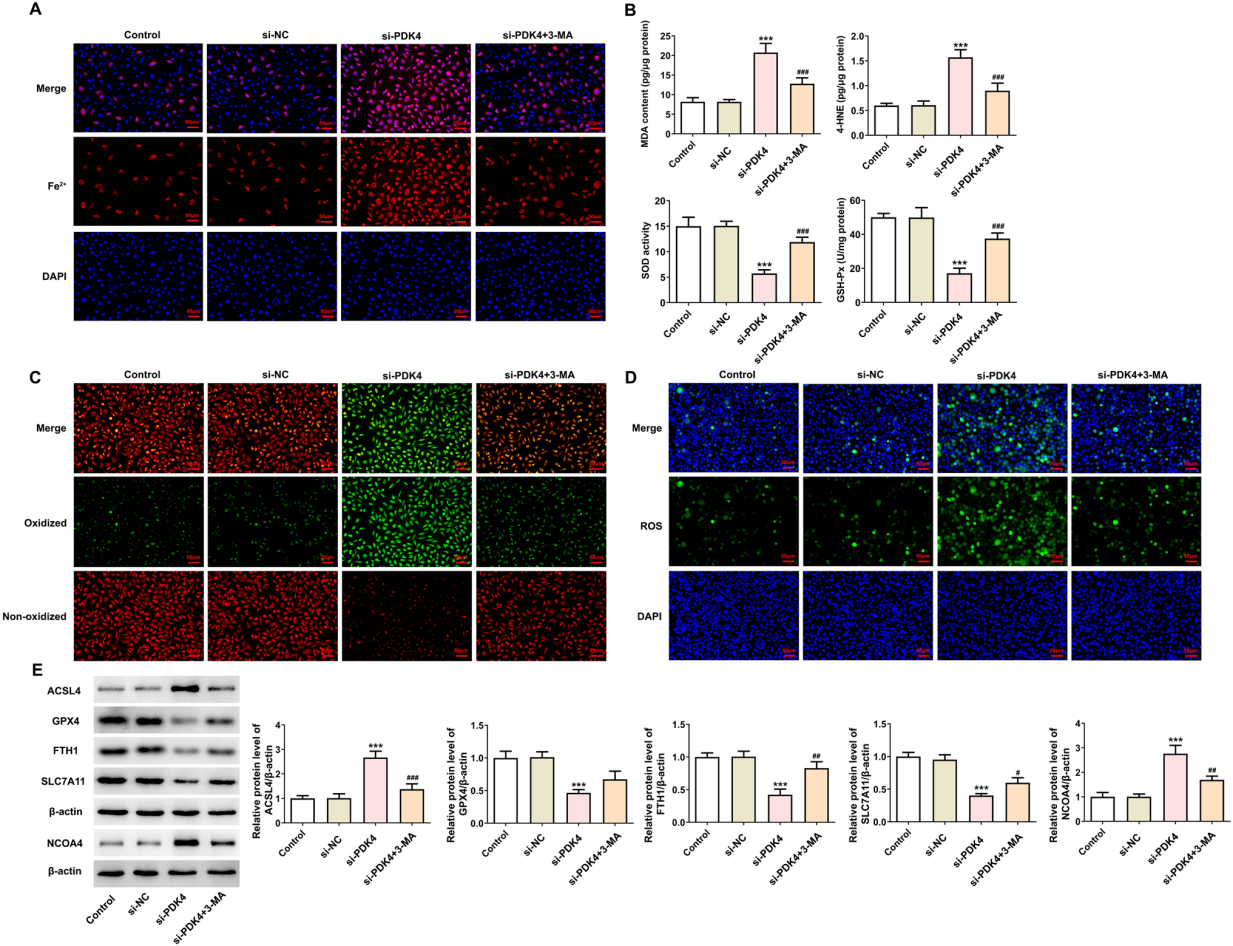
PDK4 silence activates autophagy-dependent ferroptosis in MCF-7 cells. **A** Iron Assay Kit evaluated total iron content. **B** Corresponding assay kits assessed lipid peroxidation level. **C** C11-BODIPY (591/581) staining estimated lipid ROS generation. **D** DCFH-DA staining estimated intracellular ROS production. **E** Western blotting examined the protein levels of ferroptosis-associated proteins. Data are presented as mean ± SD. n = 3. ^***^P < 0.001 vs. si-NC. ^#^P < 0.05, ^##^P < 0.01, ^###^P < 0.001 vs. si-PDK4

Autophagy has been considered as a potential contributor to ferroptosis in cancer cells. To explore whether PDK4 also impacted ferroptosis in breast cancer via mediating autophagy, autophagy inhibitors 3-MA and CQ, were utilized. Through corresponding kits, total iron content was discovered to be increased by PDK4 silencing and then be reduced by 3-MA or CQ (Fig. 3A, Figure S1A). In addition, PDK4 down-regulation increased MDA, 4-HNE concentrations and decreased SOD, GSH-Px concentrations, which were then reversed by treatment with 3-MA (Fig. 3B). Besides, the experimental results from C11-BODIPY (591/581) staining and DCFH-DA staining manifested that 3-MA significantly inhibited lipid and intracellular ROS activities, which were both intensified in PDK4-silencing MCF-7 cells (Fig. 3C, D). Also, PDK4 insufficiency up-regulated ACSL4 and NCOA4 protein levels whereas down-regulated GPX4, FTH1 and SLC7A11 protein levels in MCF-7 cells, which were all restored by autophagy inactivation (Fig. 3E, Figure S1B). To sum up, PDK4 interference might promote ferroptosis via autophagy in MCF-7 cells.

### Interference with PDK4 activates ASK1/JNK signaling in MCF-7 cells

**Fig. 4:**
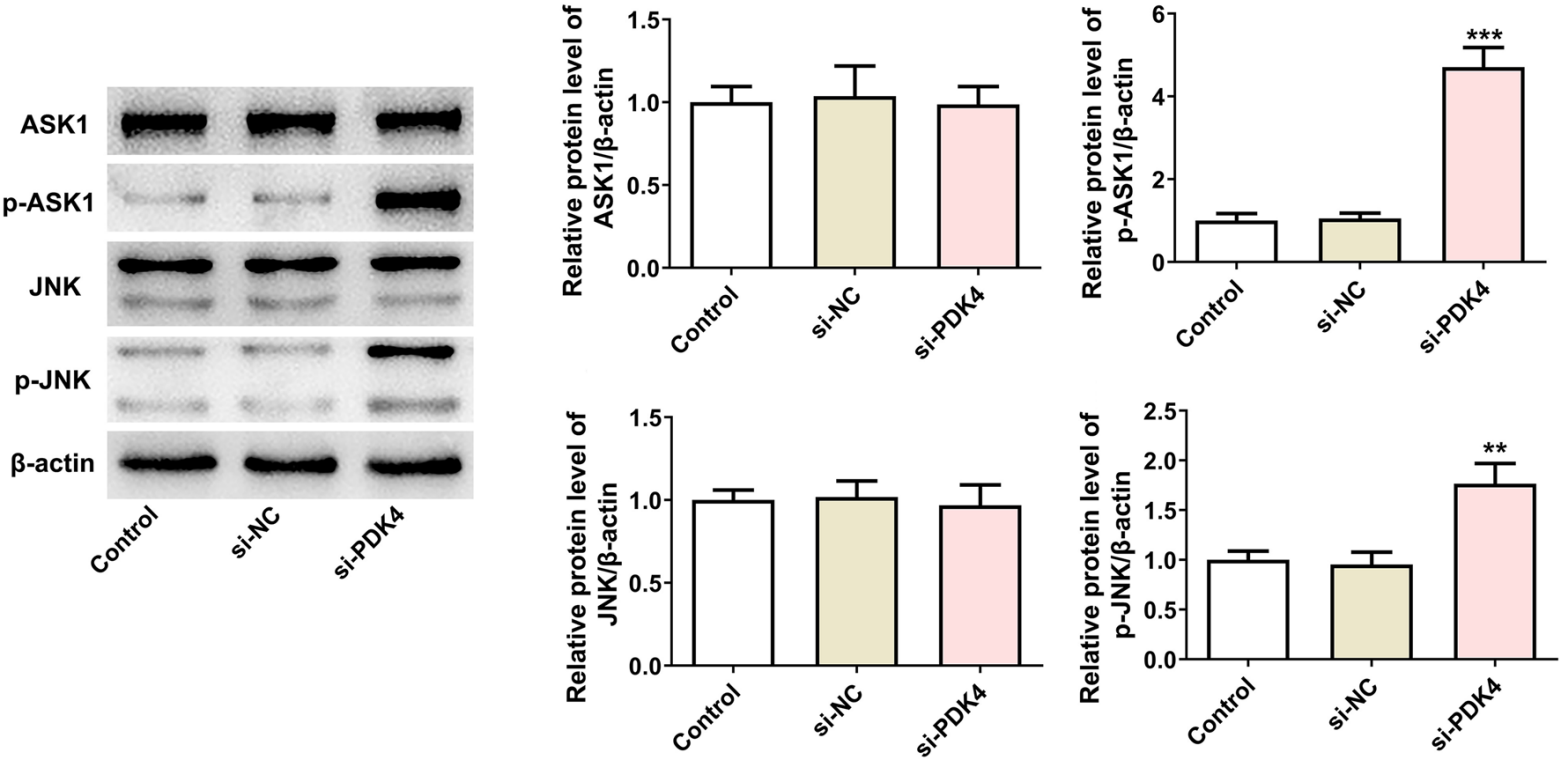
Interference with PDK4 activates ASK1/JNK signaling in MCF-7 cells. Western blotting examined the protein levels and phosphorylated protein levels of ASK1 and JNK. Data are presented as mean ± SD. n = 3. ^**^P < 0.01, ^***^P < 0.001 vs. s-NC

At the same time, through western blotting, the down-regulation of PDK4 was intriguingly noted to enhance the phosphorylated protein levels of ASK1 and JNK in MCF-7 cells (Fig. 4), implying that PDK4 silence might function as an activator of ASK1/JNK signaling in breast cancer cells.

### PDK4 downregulation promotes autophagy-dependent ferroptosis in MCF-7 cells via ASK1/JNK signaling

**Fig. 5:**
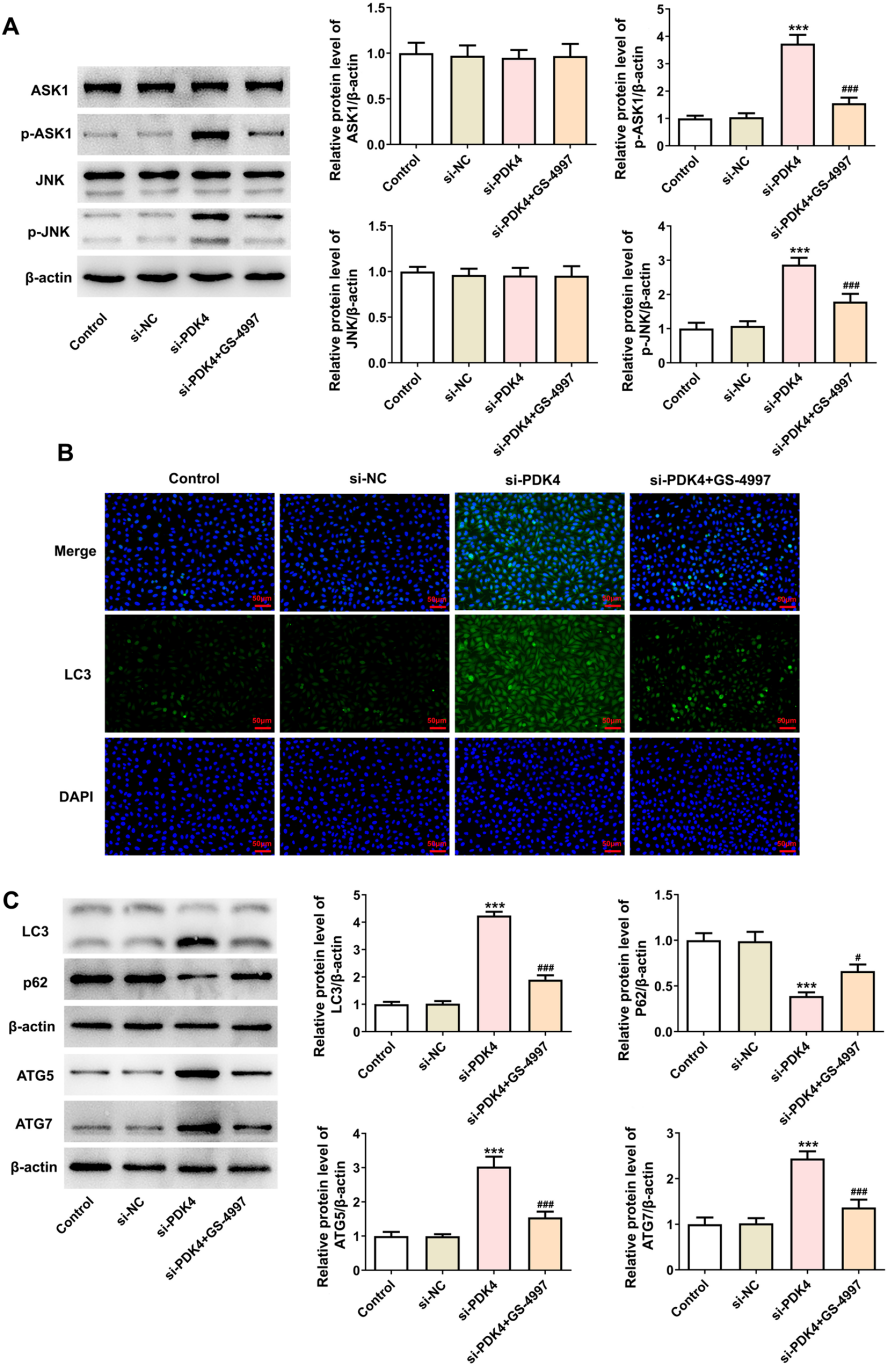
PDK4 interference promotes the autophagy in MCF-7 cells via ASK1/JNK signaling. **A** Western blotting examined the protein levels and phosphorylated protein levels of ASK1 and JNK. **B** Immunofluorescence staining appraised LC3 expression. **C** Western blotting tested the protein levels of autophagy-associated proteins. Data are presented as mean ± SD. n = 3. ^***^P < 0.001 vs. si-NC. ^#^P < 0.05, ^###^P < 0.001 vs. si-PDK4

**Fig. 6:**
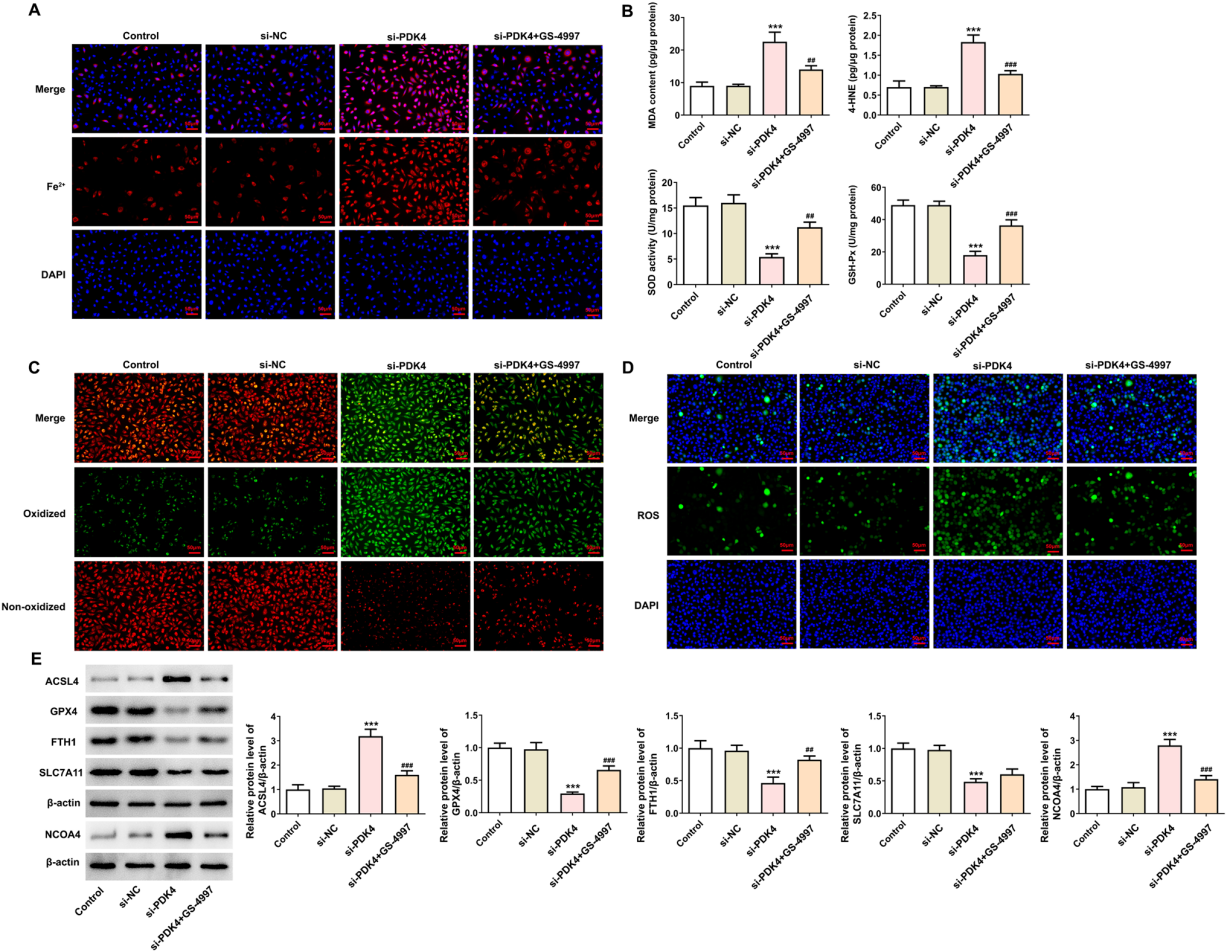
PDK4 interference promotes ferroptosis in MCF-7 cells via ASK1/JNK signaling. **A** Iron Assay Kit evaluated total iron content. **B** Corresponding assay kits assessed lipid peroxidation level. **C** C11-BODIPY (591/581) staining estimated lipid ROS generation. **D** DCFH-DA staining estimated intracellular ROS production. **E** Western blotting examined the protein levels of ferroptosis-associated proteins. Data are presented as mean ± SD. n = 3. ^***^P < 0.001 vs. si-NC. ^##^P < 0.01, ^###^P < 0.001 vs. si-PDK4

With the goal of substantiating the involvement of ASK1/JNK signaling in PDK4-mediated autophagy-dependent ferroptosis in breast cancer cells, ASK1 inhibitor GS-4997 was also utilized. After the treatment with GS-4997, the up-regulated phosphorylated protein levels of ASK1 and JNK in MCF-7 cells transfected with si-PDK4 were both downregulated (Fig. 5A). Meanwhile, GS-4997 decreased LC3, ATG5 and ATG7 protein levels whereas increased p62 protein level in PDK4-silenced MCF-7 cells (Fig. 5B, C). Additionally, the increased levels of total iron, MDA, 4-HNE and decreased activities of SOD and GSH-Px in PDK4-silenced MCF-7 cells were both abolished by GS-4997 (Fig. 6A, B). It was observable from C11-BODIPY (591/581) staining and DCFH-DA staining that PDK4 knockdown increased the production of lipid and intracellular ROS, which was decreased again by GS-4997 (Fig. 6C, D). Also, treatment with GS-4997 evidently downregulated ACSL4 and NCOA4 protein levels and upregulated GPX4, FTH1 and SLC7A11 protein levels in MCF-7 cells transfected with si-PDK4 (Fig. 6E). Taken together, inhibition of PDK4 might activate ASK1/JNK signaling to promote autophagy-dependent ferroptosis in breast cancer cells.

## Discussion

Breast cancer is a complicated and heterogeneous disease characterized by the accumulation of multiple molecular alterations. Numerous reports have evidenced that PDK4 is aberrantly expressed in human malignancies, such as gastric cancer (Zhang, et al. 2022), liver cancer (Si et al. 2023), ovarian cancer (Wang et al. 2019) and so on. Importantly, PDK4 expression has been revealed to be up-regulated in breast cancer tissues (Guda et al. 2018) and PDK4 is involved in Warburg effect, drug resistance and aggressive cellular behaviors in breast cancer (Guda et al. 2018; Dwyer, et al. 2023; Huang et al. 2021; Walter et al. 2015). Consistently, increased PDK4 expression in breast cancer cells was also confirmed in our present work. Functionally, silencing of PDK4 induced autophagy and ferroptosis in breast cancer cells. Mechanistically, activation of ASK1/JNK signaling mediated by PDK4 silence might promote autophagy-dependent ferroptosis in breast cancer cells.

Autophagy represents a core molecular pathway devoted to degrade and recycle cellular components that is regulated by the ATG proteins, dependent on which cells are capable of maintaining cellular homeostasis in response to various types of stress conditions (Klionsky et al. 2021). LC3 is known as an autophagy marker and the conversion of unconjugated LC3 (LC3-I) to conjugated LC3 (LC3-II) indicates the status of autophagy activation (Schaaf et al. 2016). p62 is a prototype autophagic substrate which accumulates when autophagy is impaired (Lamark et al. 2017). Autophagy has been reported to be closely correlated with the malignant transformation of cancer cells (Onorati et al. 2018; Li et al. 2020). Existing study has demonstrated that autophagy may play the multifaceted role in breast cancer tumorigenesis and metastasis (Wu and Sharma 2023; Niklaus, et al. 2021). Notably, PDK4 regulates autophagy to participate in vascular calcification (Ma et al. 2020) and non-small cell lung cancer (Zhang et al. 2021). Here, our experimental results illustrated that LC3 and LC3B expression, LC3-II/I, ATG5 and ATG7 protein levels were increased, p62 expression and protein level were reduced after PDK4 was depleted in MCF-7 cells, suggesting that PDK4 interference might stimulate autophagy in breast cancer cells.

Intriguingly, autophagy is engaged in the regulation of iron storage and ROS, therefore being deemed as an executioner of ferroptosis and the positive correlation between intracellular autophagy and ferroptosis sensitivity has been displayed in cancers (Liu et al. 2021b). Ferroptosis is an unconventional pattern of regulated necrotic cell death characterized by iron deposition, oxidative stress, lipid repair imbalance, and mitochondria-specific pathological manifestations (Xie et al. 2016). The imbalance between lipid peroxidation generation and clearance may trigger ferroptosis and iron overload may induce and amplify lipid peroxides (4-HNE and MDA) through producing ROS by Fenton reaction in turn (Rochette, et al. 2022). SOD and GSH-Px are enzymatic antioxidants modulating the iron-dependent lipid peroxidation. Our results, together with the published data, corroborated that the down-regulation of PDK4 increased total iron, MDA, 4-HNE, lipid and intracellular ROS levels and reduced SOD, GSH-Px concentrations, accompanied with upregulated ACSL4 and NCOA4 (ferroptosis-promoting protein) protein levels and downregulated GPX4, FTH1 and SLC7A11 (ferroptosis-inhibiting protein) protein levels in MCF-7 cells, which were all partly offset by autophagy inhibitor 3-MA. As expected, CQ pretreatment also reverted the impacts of PDK4 silencing on total iron, ACSL4, NCOA4, GPX4, and SLC7A11 protein levels in MCF-7 cells. Consequently, it was concluded that PDK4 inhibition might also drive ferroptosis dependent on autophagy in breast cancer cells.

ASK1, a member of the MAPK kinase kinase family, is activated in response to a myriad of stress such as calcium influx, endoplasmic reticulum (ER) stress, ROS, and extracellular inflammatory signals and subsequently phosphorylates and initiates the JNK and p38 MAPK pathways to trigger cellular responses such as cell death and inflammation (Ogier et al. 2020). Meanwhile, PDK4 inactivates the ASK1/P38 pathway to reduce neuronal apoptosis in early brain injury after subarachnoid hemorrhage (Gao et al. 2022) and modulates JNK pathway to drive metastasis in bladder cancer (Lee, et al. 2022). In the current research, ASK1/JNK signaling was activated by PDK4 silence, manifested by the upregulated phosphorylated protein levels of ASK1 and JNK in MCF-7 cells. Moreover, targeting ASK1/JNK signaling is well-established to effectively activate autophagy in breast cancer (Zhao et al. 2019). Further, ASK1/JNK signaling is activated by ROS produced by iron (Nakamura et al. 2019). Through investigation, ASK1 inhibitor (GS-4997) was also proved to countervail the intensified autophagy and ferroptosis of MCF-7 cells transfected with si-PDK4.

## Conclusion

Briefly, our findings supported the speculation that PDK4 might inactivate ASK1/JNK signaling to suppress autophagy-dependent ferroptosis in breast cancer. Based on our findings, we proposed a novel anti-breast cancer mechanism mediated by PDK4.

### Supplementary Information

Below is the link to the electronic supplementary material.

Supplementary Supplementary Figure 1 PDK4 interference activates autophagy-dependent ferroptosis in MCF-7 cells. (A) Iron Assay Kit evaluated total iron content. (B) Western blotting examined the protein levels of ferroptosis-associated proteins. Data are presented as mean ± SD. n = 3. ***P<0.001 vs. si-NC. ##P<0.01, ###P<0.001 vs. si-PDK4 file1 (TIF 71281 KB)

## Data Availability

We state that the data will not be shared since all the raw data are present in the figures included in the article.
